# Varicella-Zoster Viruses Associated with Post-Herpetic Neuralgia Induce Sodium Current Density Increases in the ND7-23 Nav-1.8 Neuroblastoma Cell Line

**DOI:** 10.1371/journal.pone.0051570

**Published:** 2013-01-31

**Authors:** Peter G. E. Kennedy, Paul Montague, Fiona Scott, Esther Grinfeld, G. H. Ashrafi, Judith Breuer, Edward G. Rowan

**Affiliations:** 1 Institute of Infection, Immunity and Inflammation, College of Medical, Veterinary and life Sciences, University of Glasgow, Glasgow, United Kingdom; 2 University College London Division of Infection & Immunity, United Kingdom; 3 Kingston University London, Kingston upon Thames, United Kingdom; 4 Strathclyde Institute of Pharmacy and Biomedical Sciences, University of Strathclyde, Glasgow, United Kingdom; University of Pittsburgh School of Medicine, United States of America

## Abstract

Post-herpetic neuralgia (PHN) is the most significant complication of herpes zoster caused by reactivation of latent Varicella-Zoster virus (VZV). We undertook a heterologous infection *in vitro* study to determine whether PHN-associated VZV isolates induce changes in sodium ion channel currents known to be associated with neuropathic pain. Twenty VZV isolates were studied blind from 11 PHN and 9 non-PHN subjects. Viruses were propagated in the MeWo cell line from which cell-free virus was harvested and applied to the ND7/23-Nav1.8 rat DRG x mouse neuroblastoma hybrid cell line which showed constitutive expression of the exogenous *Nav* 1.8, and endogenous expression of *Nav* 1.6 and *Nav* 1.7 genes all encoding sodium ion channels the dysregulation of which is associated with a range of neuropathic pain syndromes. After 72 hrs all three classes of VZV gene transcripts were detected in the absence of infectious virus. Single cell sodium ion channel recording was performed after 72 hr by voltage-clamping. PHN-associated VZV significantly increased sodium current amplitude in the cell line when compared with non-PHN VZV, wild-type (Dumas) or vaccine VZV strains ((POka, Merck and GSK). These sodium current increases were unaffected by acyclovir pre-treatment but were abolished by exposure to Tetrodotoxin (TTX) which blocks the TTX-sensitive fast Nav 1.6 and Nav 1.7 channels but not the TTX-resistant slow Na*v* 1.8 channel. PHN-associated VZV sodium current increases were therefore mediated in part by the Nav 1.6 and Nav 1.7 sodium ion channels. An additional observation was a modest increase in message levels of both *Nav*1.6 and *Nav*1.7 mRNA but not *Nav* 1.8 in PHN virally infected cells.

## Introduction

VZV is a neurotropic human alpha herpes virus that causes varicella (chickenpox) as a primary infection following which it becomes latent in neurons in the dorsal root ganglia (DRG) and trigeminal ganglia (TG) [1], [2]. After a variable latent period, the virus may undergo reactivation to cause herpes zoster (shingles) which is a painful vesicular rash occurring along the distribution of one or more sensory dermatomes. The most important complication of zoster is post-herpetic neuralgia (PHN) which causes severe pain in the affected dermatome which persists for more than 3 months after the rash, and occurs in about 50% of individuals over 60 years [3]. Although it has a high morbidity, the mechanism causing PHN remains unknown, its occurrence cannot be predicted at the time of zoster and its treatment is still highly unsatisfactory and generally ineffective [4].

Both host and viral factors are likely to be important in determining the incidence and mechanism of PHN. VZV DNA was reported in several studies as persisting in peripheral blood mononuclear cells (PBMCs) in PHN patients compared with zoster patients without PHN [5], [6], [7] indicating a role for persistent viral infection in the pathogenesis of PHN, though another study could not detect VZV DNA or RNA in PBMCs of patients with PHN [8]. It is also a possibility that different VZV strains may have variable determinants to produce PHN. Patients with PHN also showed a significant improvement on a quantitative pain severity scale after treatment with the acyclovir [9], suggesting a possible role of active virus replication in producing or contributing to PHN. A key question is how the virus exerts its effects on neuronal cells.

Several lines of evidence suggest that VZV may cause PHN through a direct effect on voltage-gated sodium ion channels (VGSCs). The latter are located on the neuronal plasma membrane and mediate the influx of sodium ions into the cell as a result of membrane depolarisation [10, 11)] The sodium channel α subunits are large polytopic transmembrane proteins that are highly conserved through evolution. Duplication of these subunit genes has resulted in the evolution of nine functional genes distributed between two gene clusters (*Nav* 1.1, 1.2, 1.3, 1.7), (*Nav* 1.5, 1.8, 1.9) with *Nav*1.6 and *Nav*1.4 on single loci) encoding corresponding active ion channels which have different tissue distributions and biophysical properties [11], [12]. There is abundant evidence that altered sodium ion channel activity in peripheral neurons is associated with the development of inflammatory and neuropathic pain due to neuronal hyperexcitability [10], [13], [14]. There are two general classes of sodium ion channels, those, such as Nav 1.3, Nav 1.6 and Nav1.7 the activity of which is blocked by, that is sensitive to, the neurotoxin tetrodotoxin (TTX-s), and others, such as Nav1.8 and Nav 1.9, that are resistant to TTX (TTX-r) [10], [13]. While there is good evidence that both classes of sodium ion channels are associated with neuropathic pain and may show increased protein and/or RNA message levels in such conditions [13], [15], there remain many questions concerning the role of sodium ion channels in neuropathic pain, in particular in PHN. Any observed alteration in the activity of sodium ion channels may be relevant to the development of neuropathic pain. Both TTX-r and TTX-s sodium ion channels are found in DRG neurons [11], [13], which is the site of latent VZV [16]. It has been shown that HSV-1, another human herpes virus, causes loss of excitability of sodium ion channels in cultured rat DRG neurons through selective internalisation from the plasma membrane [17]. Recently, it has been reported that wild-type pseudorabies virus can also modulate electrical activity in cultured sympathetic rat neurons [18]. Moreover, in a rat model of VZV-induced PHN it was shown that DRG neurons had elevated levels of the sodium ion channel proteins Nav 1.3 and Nav 1.8, and the VZV-induced neuropathic responses were reversed by sodium ion channel blockers [15]. There have also been several other reports using the rat PHN model in which both wild-type virus and clinical isolates have been shown to induce mechanical allodynia and thermal hyperalgesia, the two key behavioural measurements of pain [19]–[23]. Further, circulating virus could conceivably have a phenotypic effect such as altering the neurophysiological and/or biochemical phenotype of neurons in the affected peripheral ganglia that may be relevant to the development of neuropathic pain. Moreover, drugs such as carbamazepine and lidocaine, which are known to have sodium ion channel blocking activity, have some efficacy in relieving the pain of PHN [24]. In this study we tested the hypothesis that VZV obtained from individuals who had subsequently developed PHN would have a differential effect on the generation of sodium ion channel currents *in vitro* compared with VZV isolates from individuals who did not develop PHN. This was tested by single cell patch clamping of ND7/23-Nav 1.8 cells following infection with VZV isolated from PHN and non-affected individuals.

## Materials and Methods

### Ethics Statement

Ethical approval to obtain and study human material was obtained from East London and the city research ethics committee LREC R&WF2002/38.

### Viruses and cell lines

20 different VZV isolates, comprising 11 PHN samples and 9 non-PHN samples were studied [25], [26] (Table 1). PHN was defined as persistent dermatomal pain of- >3 by the ZBPI at 3 months following an episode of herpes zoster. Non-PHN was defined as absence of pain by 6 weeks after the onset of rash. In addition, wild-type VZV (Dumas strain) as a control for the vaccine isolates varivax (Oka/Merck) varilrix (GSK) were obtained. The melanoma cell line MeWo [27] was used for initial infection and propagation of VZV was maintained in 10% DMEM. The mouse neuroblastoma x rat DRG hybrid cell line ND7/23-Nav1.8 propagated in 10% DMEM [28] harbours exogenous copies of the mouse *Scn*10 cDNA encoding the Nav1.8 TTX-resistant slow sodium ion channel was used for sodium ion channel measurements in single cell patch-clamp experiments. Generation of MeWo cell lines infected with the PHN and non-PHN VZV isolates are described elsewhere [25].

**Table 1:** Post herpetic neuralgia (PHN)-associated and non-PHN associated viruses used in study.

Sample	PHN	Age	Gender
S29	no	71	M
SUK57	no	40	M
S69	no	55	M
Z202	no	28	M
Z208	no	24	M
S10	no	83	M
S22	no	61	M
S45	no	64	M
S70*	no	39	M
Z132	yes	39	M
ZAP198	yes	60	M
Z201	yes	85	F
Z226	yes	72	F
S66	yes	45	M
Z134	yes	51	F
Z143	yes	68	M
Z210¶	yes	70	F
Z246+	yes	70	F
Z281	yes	46	M
Z214	yes	39	M

The sample reference numbers, age and sex of the patients are indicated.

* HIV positive.

+ Immunosuppressed and on corticosteroids.

¶ On corticosteroids.

### Preparation of cell-free virus from infected MeWo cell lines

In a T75 cm^2^ flask a mixture of 3.33×10^5^of VZV infected MeWo cells and 2.67×10^6^ of non-infected MeWo cells (1∶8 ratio) were incubated up to 72 hours or until a cytopathic effect (cpe) of around 5–30% was obtained. The cells were rinsed with PBS and scraped in the presence of 1 ml of PBS-Sucrose-Glutamate-Serum (PSGS) cryo-preservative buffer and sonicated (Sonics VibraCell) three times for 15 seconds at a setting of 20 kHz with an amplitude of 10 µM with 15 second rests. Following clarification of the supernatant by centrifugation at 1000 g for 15 mins at 4°C the Sonicated Viral Supernatants (SVS) were stored at −80°C.

### Heterologous infection of ND7/23-Nav1.8 cells

10^5^ ND7/23-Nav1.8 cells were plated into a T10cm^2^ flask and incubated overnight. The growth medium was replaced with 500 µl of SVS and 500 µl of 10% FBS/DMEM for a six hour absorption period. Following two PBS washes the “infected” cells were incubated for 72 hours prior to single cell patch clamp electrophysiology. Compared to infected human MeWo cells which show a cytopathic effect (cpe) in the form of distinct syncytia, infected rat/mouse ND7/23-Nav1.8 cells have no phenotype. Accordingly, SVS infection efficiency of this cell type was determined by RT.PCR relative to the infection obtained with the human MeWo cell line by measuring VZV ORF18 message levels. These assays suggested that the infection rate of the rat line ND7/23-Nav1.8 was around 10–25% of the human line. Using these efficiency differences between ND7/23-Nav1.8 rat cells and the human lines as guidelines, infection of the ND7/23-Nav1.8 cell line was performed on 10^5^ cells in conditions of a 10–20 fold excess compared to MeWo cells.

### End-point RT.PCR analysis of infected ND7/23-Nav1.8 cells

4×10^4^ of ND7/23-Nav1.8 cells were plated onto 13 mm well dishes and incubated overnight at 37°C. The cells were washed in PBS and overlaid with a mixture comprising 250 µl of SVS and 250 µl 10% DMEM for six hours, washed twice with PBS and incubated at 37°C for 72 hours. Total cellular RNA prepared by homogenization in RNA-Bee-(amsbio-UK) was converted to cDNA using SuperScript III (Invitrogen-UK) for end-point RT.PCR analysis. PCRs were performed on 5 ng of cDNA using RedTaq Ready Mix (Sigma-UK) against the following primer sets: Cyclophilin: Forward (5′-ACCCCACCGTGTTCTTCGAC-3′), Reverse (5′-CATTTGCCATGGACAAGATG-3′) product length 300 bps.VZV*ORF1*8 Forward (5′-TCGCTGATAAAAGCCTGTCC-3′) VZV *ORF*18 Reverse (5′-ATCATCGTTCGCGGCTATTGC-3′)product length 293 bps. *Nav*1.3 Forward (5′-CTCGAGAATCTCTTGCTGCTATCGA-3′).*Nav*1.3 Reverse (5′-AGTTCCATGGGTCACGAAGAAA-3′) product length 500 bps. *Nav*1.6 Forward (5′-CACCGGGAGGACGATGAAGACC-3′) *Nav*1.6 Reverse (5′-TGAAACATTGCCTAGGTTTACA-3′) product length 293 bps. *Nav*1.7 Forward (5′-AGATGATGAGGAAGAAGGTCCCAA-3′) *Nav*1.7 Reverse (5′-CGAAGAGCTGAAACATTGCCTA-3′) product length 500 bps. *Nav*1.8 Forward (5′-GTTGATTCCGGAGAGATCAACAGTCA-3′) *Nav*1.8 Reverse (5′-GACAGGATCACCACTATGAAGTCGA-3′) product length 500 bps. Thermal cycling parameters were an initial denaturation step of 94°C/5 mins, a core cycle comprising (94°C/1 min−55°C−65°C/1 min−72°C/1 min) for 25–35 cycles followed by a final extension of 72°C/10 mins.

All PCRs were performed in the linear amplification range and message levels corresponding to the genes of interest were expressed relative to the activity of the mouse housekeeping gene *Cycolphilin* as described elsewhere (20). PCR products were separated by TAE gel electrophoresis, visualised by ethidium bromide staining and quantified by densitometry using a UVIdocD55XD documentation system (Uvitec UK). A one-way ANOVA-Bonferroni Multiple Comparison Test was performed on the data sets to determine any statistical significance in sodium ion channel gene activity between non-infected cells and cells infected with SVSs prepared from non-PHN and PHN VZV infected MeWo cells.

### Recording of sodium ion channel activity in VZV-infected ND7/23-Nav1.8 cells

All electrophysiological procedures were carried out in a blinded fashion using coded virus isolates unknown to the operator (EGR). Infected and control T10 cm^2^ flasks were trypsinized and resuspended at 1×10^6^ cells per ml of NaCl (129 mM), KCl (3.25 mM), CaCl_2_ (2 mM), MgCl_2_ (2 mM), HEPES (10 mM), D-glucose (11 mM), TEA-Cl (20 mM), pH 7.4, 345 mOsM.

For sodium ion channel recording, ND7/23-Nav1.8 cells were voltage-clamped at room temperature (20–22°C) in the whole-cell configuration mode using a Axopatch 1D (Axon Instruments) controlled by Whole Cell Analysis Program (WCP) V4.2.0 (Dr J Dempster, University of Strathclyde) running on an IBM compatible personal computer which is used for data acquisition and pulse generation. Data were sampled at 10 kHz after being filtered at 2 kHz. Fire-polished patch pipettes were pulled from borosilicate glass capillaries (GC150F-10, Harvard Apparatus) using a DMZ Universal puller (Zeitz Instruments, Gmbh, Germany) that had resistances of 1–1.5 MΩ when filled with the following internal pipette solution: CsF (120 mM), NaCl (10 mM), HEPES(10 mM), EGTA (11 mM), TEA-Cl (10 mM), CaCl_2_ (1 mM), MgCl_2_(1 mM), pH 7.3, 325 mOsM. The initial seal resistance after establishing a whole cell patch was 2–5 GΩ and recordings were discarded if the seal resistance fell below 1GΩ during the course of the experiment. Capacitative transients were electronically cancelled and voltage errors were minimised by applying between 75–95% series resistance compensation. Linear leak currents were subtracted off-line using a P/4 subtraction protocol. Currents were scrutinized for voltage artefacts and current-voltage relationships were characteristic of appropriately clamped cells. In order to determine the tetrodotoxin (TTX) sensitivity of the currents, TTX (250 nM) was included in the external solution and currents recorded in the continued presence of the drug.

Total sodium currents (using appropriate internal and external solutions) was evoked by a 25 ms voltage step from the holding potential (−120 mV) to a command potential (−90 to +80 mV) in steps of 5 mV every second. The conductance and half-maximal activation voltages (GNa; V_½_) was calculated using the equation: GNa = INa/(Vm−Vrev), where INa is peak current, Vm the test potential and Vrev the reversal potential as determined from the individual I/V plots. The results obtained with PHN VZV were compared with non-PHN VZV infected cells and uninfected control cells using one-way ANOVA followed by a Newman-Keuls multiple comparison *post hoc* test. Having characterised the electrophysiological phenotype of human VZV isolates, the identical methodology was used to determine the effects on sodium ion channel activity of the live attenuated Merck, GSK vaccine strains and in a number of VZV isolates that had been pre-treated with acyclovir in this system. The vaccines have differences from parental/wild-type strains based on their sequences and gene expression levels so we anticipated that they would show a different phenotype from the human isolates and parental P-Oka strains.

## Results

### Relative abundance of *Nav*1.3, *Nav*1.6, *Nav*1.7 and *Nav*1.8 transcripts in infected ND7/23-Nav1.8 cells

**Figure 1:**
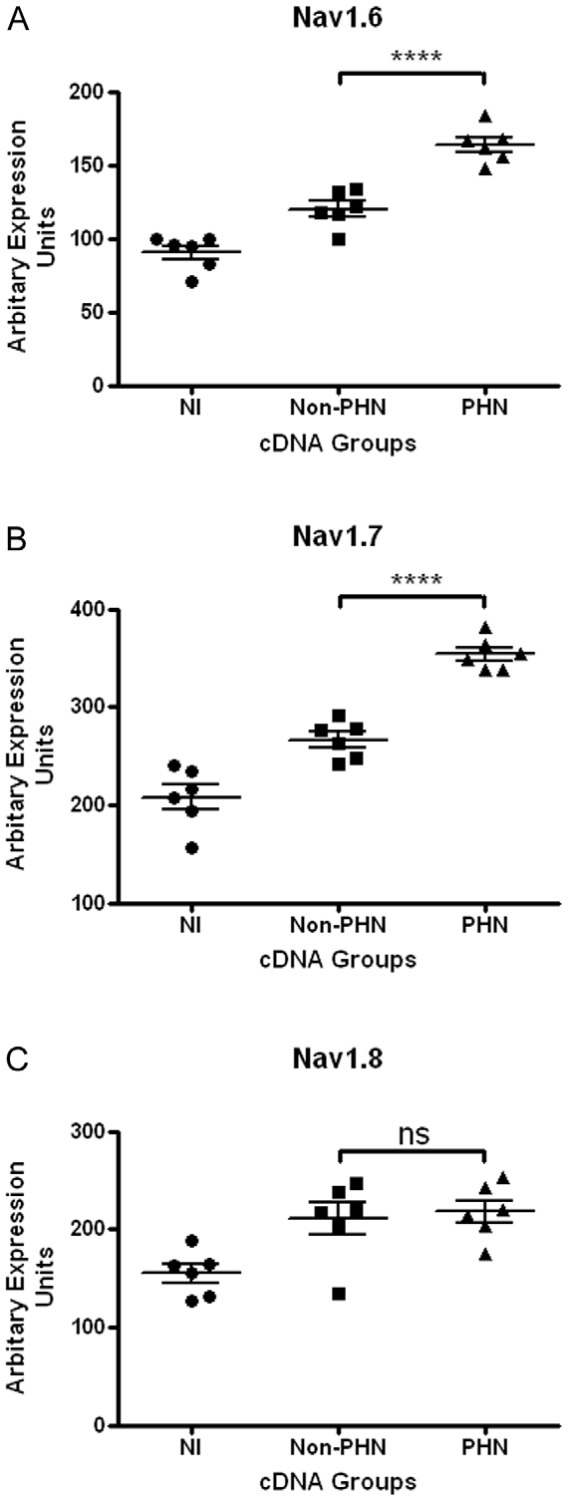
RT.PCR end-point analysis depicting expression of (A) *Nav*1.6, (B) *Nav*1.7 and (C) *Nav*1.8 genes in non-infected ND7/23-Nav1.8 cells (NI) and cells infected with SVSs prepared from non-PHN VZV isolates (Non-PHN) and PHN VZV isolates (PHN). Each grouping comprised six cDNAs representing three separate experiments. One-way ANOVA-Bonferroni Multiple Comparison Test analysis shows a modest but statistically significant increase (**** P<0.0001) in the transcriptional activity of the *Nav*1.6 and *Nav*1.7 genes between the non-PHN and PHN groupings and contrasted to *Nav*1.8 mRNA levels which remained essentially invariant (ns) between the non-PHN and PHN groups.

**Figure 2:**
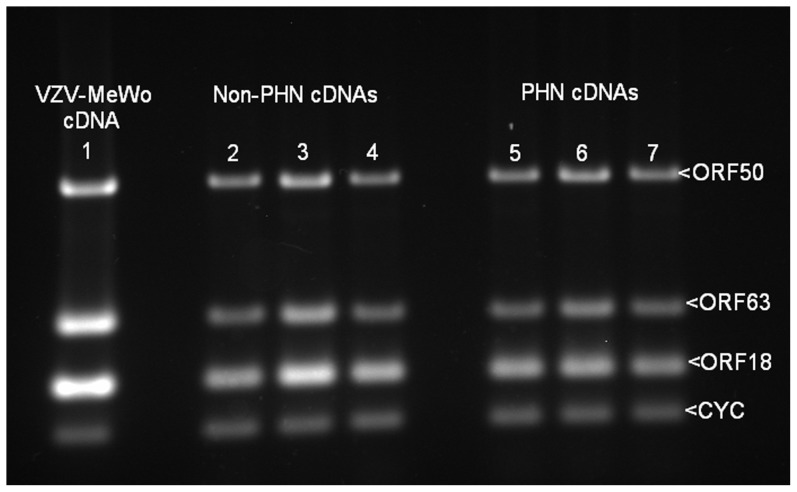
VZV gene 63, 18, and 50 transcripts in infected ND7/23 1.8 cells. End point PCR analysis depicting expression of the VZV genes ORF63_IE_, ORF18_E_ and ORF50_L_ relative to the housekeeping gene cyclophilin (CYC) in; VZV infected MeWo cells (Lane1), ND7/23-Nav1.8 cells infected with SVSs prepared from three non-PHN (Lanes 2–4) and three PHN (Lanes 5–7) MeWo cell lines.

The heterologous Na7/23-Nav 1.8 cell line was chosen primarily because it is known to express several types of sodium ion channel genes and had been stably transfected to express the *Nav* 1.8 TTX-R gene [28] which were required for this study. Though VZV grows productively only in human and primate cells, primary human fetal cells were not available to us and the absence of a cytopathic effect in the ND7/23-Nav 1.8 cells was a positive advantage in terms of avoiding observer bias in the selection of productively infected cells. Relative viral infection efficiency was assessed by estimating VZV *ORF*18 message levels by end-point RT.PCR revealed viral load heterogeneity presumably reflecting variability in both SVS titre values and heterologous infection efficiency. Six non-PHN and six PHN samples with a comparable *ORF*18 signal in addition to six non-infected controls were selected to examine relative expression of the *Nav*1.3, *Nav*1.6, *Nav*1.7 and *Nav*1.8 genes. *Nav*1.3 transcriptional activity was barely detectable in non-infected ND7/23-Nav1.8 cells consistent with its documented transcriptional activity during CNS fetal development [29] and there was no discernible change in the mRNA abundance in VZV infected cells. In contrast, message levels of the endogenous *Nav* 1.6 and *Nav* 1.7 mRNAs in addition to the exogenous *Nav*1.8 message levels were readily detectable in non-infected cells. Accordingly, end–point RT.PCR analysis was performed on three different data sets of each cDNA grouping to monitor changes in expression of these genes. A one-way ANOVA-Bonferroni Multiple Comparison Test was performed on the data sets identified a modest but statistically significant increase in the transcriptional activity of the *Nav*1.6 and *Nav*1.7 genes between the non-PHN and PHN groupings and contrasted to the *Nav*1.8 mRNA abundance levels which remained essentially invariant between the non-PHN and PHN groups (Figure 1). All three classes of VZV transcripts (immediate-early, early and late) as represented by *ORF*63, *ORF*18 and *ORF*50 respectively could be qualitatively detected in the infected cell line after 72 hr by RT.PCR (Figure 2), though viral proteins were absent.

### Sodium ion channel activity in PHN and non-PHN infected ND7/23-Nav1.8 cell line

Cell free SVSs prepared from MeWo cell lines infected with VZV isolates from 11 PHN and 9 non-PHN patients (Table 1) were used to infect the ND7/23-Nav1.8 cell line [28] were subjected to single cell patch clamp analysis. A prominent, transient inward sodium current was evoked from the control ND7/23-NaV1.8 cell line following brief depolarisations from negative holding potentials. Holding cells at negative membrane potentials, in the range of −120 mV, ensured that steady-state sodium ion channel inactivation was negligible and that the maximum number sodium ion channels were available during the pulse protocol.

In untreated control cells a series of 5 mV step depolarisations from a holding potential (V_hold_) of −120 mV to command voltages more positive than −50 mV elicited inactivating inward sodium ion channel currents that peaked around −10 mV and reversed direction around 42±1 mV *i.e*. close to the predicted sodium equilibrium potential. Conventional current density (pA/pF) versus membrane potential plots for control sodium ion currents showed a peak current density of −115.6±10.8 pA/pF with half maximal activation (V_½ control_) occurring at −23.5±1.3 mV; n = 41.

In cells exposed to non-PHN SVSs, a prominent transient inward sodium ion current was also evoked following brief depolarisations from negative holding potentials. The inward sodium ion current also peaked around −10 mV and reversed around +40.7±0.7 mV. Current density (pA/pF) versus membrane potential plots for sodium ion currents treated with non-PHN VZV isolates had a peak current density of −130.6±8.9 pA/pF with half maximal activation (V_½ nonPHN_) occurring at −22.7±0.9 mV; n = 86). These data sets were not significantly different from control (p>0.05).

**Figure 3:**
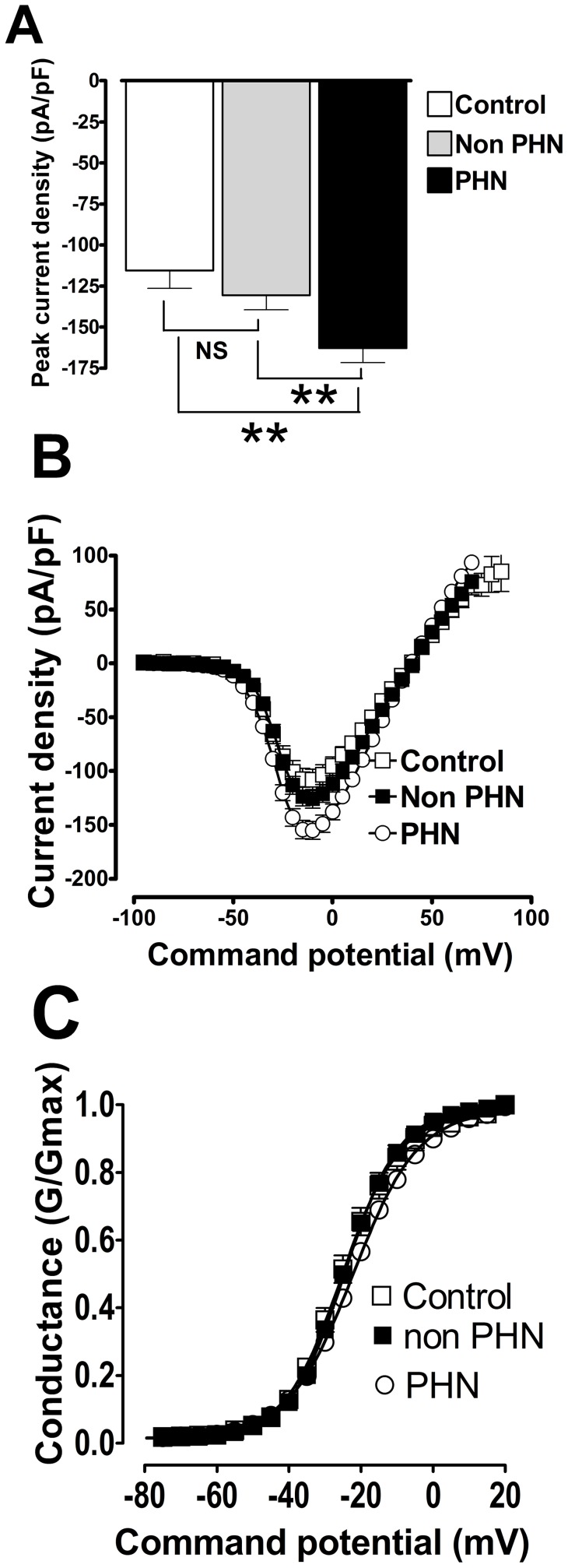
VZV isolates implicated in post herpetic neuralgia significantly increase sodium current density in ND7-23 1.8 cells. (A) Comparison of peak current densities in sham treated control cells (solid black bar), cells exposed to VZV isolates not implicated in post herpetic neuralgia (non PHN; solid grey bar) and cells exposed to VZV isolates implicated in post herpetic neuralgia (PHN, open bar) (**p<0.01, Analysis of Variance Newman-Keuls multiple comparison *post hoc* test). (B) Conventional current density verses membrane potential plots for sodium currents from sham treated control cells (open squares) and cells exposed to viral isolates non-PHN (black squares) and PHN VZV isolates (open circles). Data was generated using the following protocol: V_Hold_ = −120 mV to +100 mV depolarized in 5 mV increments for 25 ms every 1 sec. (C) Data from (B) represented as a conductance plot. Lines were generated and fitted using a standard Boltzman function that yielded similar half maximal activation voltages (V_½ control_  = −23.5±1.3 mV; V_½ nonPHN_  = −22.7±0.9 mV and V_½ PHN_  = −22.9±0.7 mV) showing that VZV isolates do not significantly alter the voltage-dependence of sodium channel activation.

In cells exposed to PHN SVSs, a prominent transient inward sodium ion current was also evoked by brief depolarisations from negative holding potentials. The inward sodium ion current peaked around −10 mV and reversed around +40±0.5 mV; the reversal potential was not significantly different from control or non-PHN cells (p>0.05). Current density (pA/pF) versus voltage plots for sodium ion currents treated with PHN VZV isolates had a peak current density of −162.9±8.7 pA/pF with half maximal activation (V_½ PHN_) occurring at −22.9±0.7 mV; n = 205). Current density versus membrane potential plots for control, non-PHN and PHN are shown in Figure 3; statistical comparisons of the peak current densities of PHN versus non-PHN and control show that they are significantly different (p<0.01). However, the V_½_ for sodium ion current activation was not significantly different from control samples. Hence, PHN appears to increase current density without altering the activation kinetics of the sodium ion channel. This increase in current density cannot be accounted for by an increase in cell size following exposure to VZV isolates as cell capacitance was not significantly changed (control, 30±2.6 pF; non-PHN, 28.2±0.9 pF and PHN, 28.0±0.81 pF).

### Sodium ion channel activity in VZV vaccine strains

**Figure 4:**
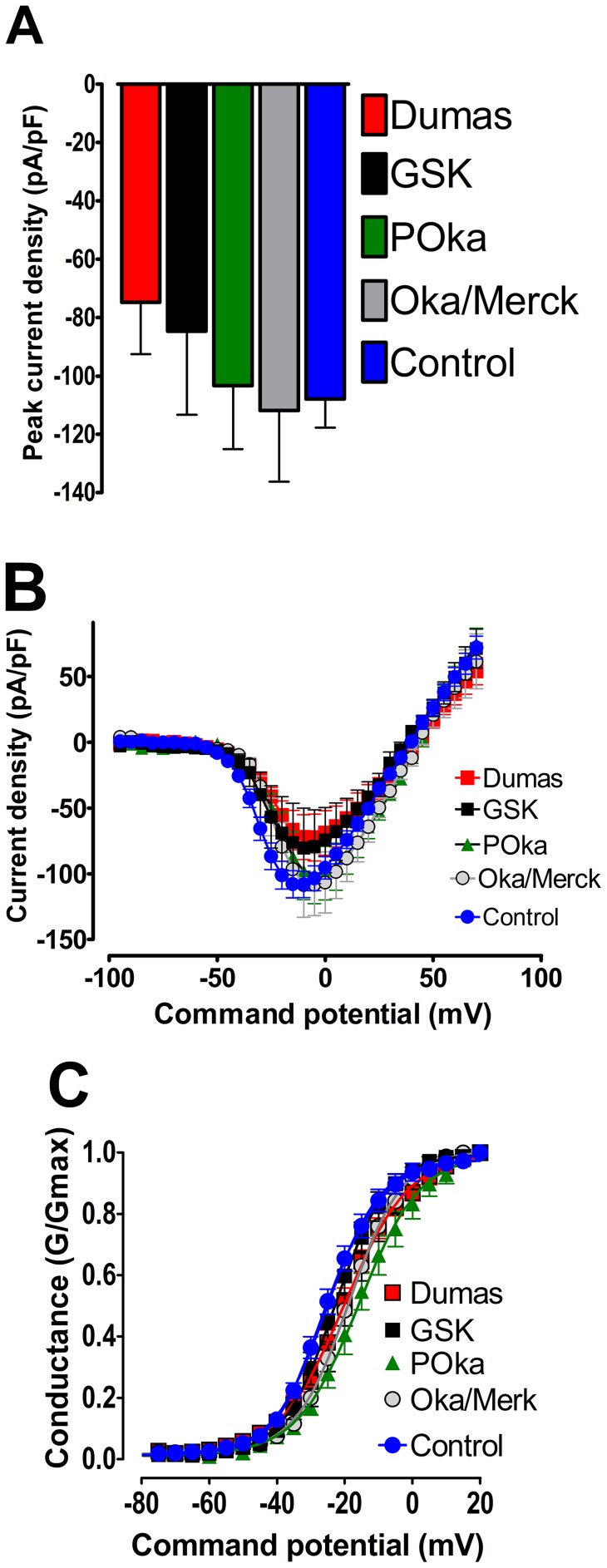
The lack of effect of VZV vaccine strains on sodium currents expressed in ND7/23 1.8 cells. (A) Comparison peak current density in cells exposed to Dumas vaccine strain (solid red bar), GSK vaccine strain (solid black bar), POka vaccine strain (solid green bar), Oka/Merk (solid grey bar) and control (solid blue bar) (p>0.05, Analysis of Variance Newman-Keuls multiple comparison *post hoc* test. (B) conventional current density verses membrane potential plots for sodium currents from cells exposed to VZV vaccine strains: Dumas vaccine strain (red squares), GSK vaccine strain (black squares) POka vaccine strain (green triangles), Oka/Merk (grey circles) and control(blue circles). Data was generated using the following protocol: V_Hold_  = −120 mV to +100 mV depolarized in 5 mV increments for 25 ms every 1 sec. (C) Data from (B) represented as a conductance plot. Lines were generated and fitted using a standard Boltzman function that yielded similar half maximal activation voltages (V_½ Dumas_  = −20.0±1.7 mV;V_½ GSK_  = −22.6±1.9 mV; V_½ POka_  = −15.0±2.9 mV; V_½ Oka/Merk_  = −17.8±1.7 mV; V_½ control_  = −21.3±2.5 mV) showing that VZV vaccine strains do not significantly alter the voltage-dependence of sodium channel activation.

When the VZV vaccine strains varivax (Oka/Merck) and varilrix (GSK) were studied using the same methodology as for the patients' VZV isolates, it was found that the changes in sodium ion channel current density induced by both vaccines was very similar to that found with the non-PHN virus, and was therefore significantly less than for PHN VZV. For example, in cells exposed to VZV wild-type (Dumas) or vaccine strains (POka, Merck and GSK) the sodium ion currents elicited by brief depolarisations were qualitatively similar to those in control cells (Figure 4) and none of the measured parameters were statistically different from controls. The inward sodium ion current peaked around −10 mV (Dumas, −74.8±17.7 pA/pF; POka, −103.4±17.2 pA/pF; Merck −111.8±24.48 pA/pF and GSK −84.7±28.6 pA/pF; n≥6) and reversed around +40 mV).

### Sodium ion channel activity following treatment with acyclovir and TTX on selected PHN VZV isolates

**Figure 5:**
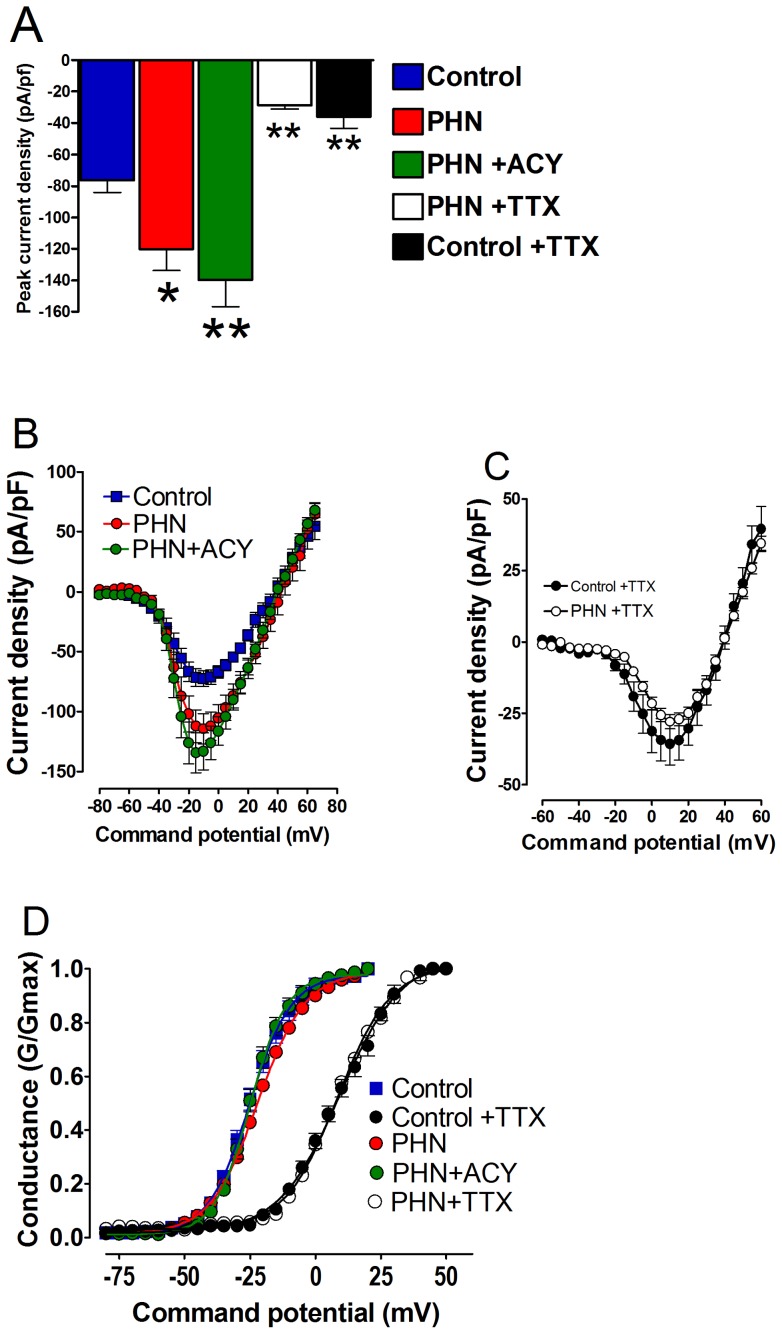
Pre-treatment with acyclovir does not prevent the TTX sensitive increase in sodium current density in ND7/23 1.8 cells induced by prior exposure to VZV isolates implicated in PHN. (A) Comparison peak current density in sham treated control cells (solid blue bar), cells exposed to VZV isolates implicated in post herpetic neuralgia (PHN, solid red bar), cells exposed to PHN VZV isolates and subsequently treated with acyclovir (solid green bar), the effect of TTX on cells exposed to PHN VZV isolates (open bar) and the effect of TTX on sham treated control cells (solid black bar) (sham treated control vs PHN *  = p<0.05; sham treated control vs PHN +ACY **  = p<0.01; PHN vs PHN+TTX **  = p<0.01, and sham treated control vs sham treated control +TTX **  = p<0.01; Analysis of Variance Newman-Keuls multiple comparison *post hoc* test. (B) and (C) Conventional current density verses membrane potential plots for sodium currents from cells exposed to PHN VZV isolates vaccine strains: sham treated control cells (blue squares) and sham treated control cells following exposure to TTX (black circles); cells exposed to PHN viral isolates in the absence (red circles) and presence of TTX(open circles); and cells exposed to PHN viral isolates that had been pre-treated with acyclovir (green circles). Data was generated using the following protocol: V_Hold_  = −120 mV to +100 mV depolarized in 5 mV increments for 25 ms every 1 sec. (D) Data from (B) and (C) represented as conductance plots. Curves were generated and fitted using a standard Boltzman function. The Boltzman curves generated from data set (B) yielded similar maximal activation voltages (V_½ control_  = −23.9±1.6 mV; V_½ ACY_  = −20.9±2.9 mV; V_½__PHN_  = −22.1±2.3 mV) and similarly curves from data set (C) yielded similar half maximal activation voltages (V_½ control+TTX_  = +9.2±1.9 mV; V_½ PHN+TTX_  = 9.5±1.2 mV.

In cells exposed to PHN-VZV SVSs isolates that were treated with acyclovir the increase in sodium current density was still present when compared to treatment matched controls (control, −76.2±7.8 pA/pF; PHN, −120.2±13.7 pA/pF; PHN + ACY, 139.9±17.0; control vs PHN p<0.05, control vs PHN +ACY p<0.01) Acyclovir did not significantly alter the voltage at which half maximal (V_½ ACY_) activation occurs when compared to control (V_½ control_ −23.9±1.6 mV, n = 15; V_½ ACY_ −20.9±2.9 mV, n = 20). In the presence of 250 nM TTX the sodium current density was significantly (p<0.01) reduced in both PHN-treated and control cells (PHN+TTX, −28.9±2.3 pA/pF, n = 22; control +TTX, −33.4±4.7 pA/pF, n = 14). In addition there was a significant (P<0.05) rightward shift in their respective half maximal activation (V _½PHN+TTX_ +9.2±1.9, n = 22; V_ ½ control+TTX_ +9.5±1.2, n = 14) in comparison to untreated controls (Figure 5).

## Discussion

In this study we showed that there was a consistent and significant difference between PHN-associated and non-PHN VZV isolates in terms of the neuronal electrical activity they induced *in vitro*. VZV obtained from individuals with PHN increased sodium ion current density in the ND7/23-Nav1.8 cell line compared with non-PHN VZV without altering the activation kinetics of the sodium ion channels. This increase in current density cannot be accounted for solely by an increase in cell size following exposure to VZV isolates since cell capacitance was not significantly changed. The three VZV vaccine strains (Merck, GSK and the POka parental strain) induced sodium ion channel changes that were very similar to that of non-PHN VZV which was consistent with their primary role as VZV vaccines. The results are summarized in Table 2. The PHN-associated viruses are a highly selected group of virus isolates, and all of these have been shown to be associated with PHN in patients. While it is certainly possible that some other viral strains such as Dumas VZV can cause pain in patients, those other viruses used in the study have not been shown to produce, or be selected for their ability to produce, PHN, so it is not entirely surprising that they showed different electrophysiological changes from PHN-associated VZV as they are different viruses.

**Table 2:** Trends in **(A)** sodium ion channel current densities and **(B)** sodium ion channel mRNA levels in PHN, non-PHN and non-infected ND7/23-Nav1.8 cells.

Status of ND7/23-Nav1.8 cells	A Relative changes of sodium ion channel current density		B Relative expression pattern of specific sodium ion channel genes			
	Non-treated	1TTX treated	2 *Nav*1.3	*Nav*1.6	*Nav*1.7	*Nav*1.8
Non-infected	+ + +	+	-	+ +	+ +	+ +
Non-PHN VZV	+ + +	^nd^	-	+ + +	+ + +	+ + +
	3p<0.01			3p<0.001	3p<0.001	3p>0.05
PHN VZV	+ + + + +	+	-	+ + + + +	+ + + + +	+ + +

1 250 nM tetrodotoxin treatment selectively blocks the fast acting sodium ion channels Nav1.6 and Nav1.7.nd A direct comparison of tetrodotoxin treated PHN and non- PHN cells was not done.

2 *Nav* 1.3 levels were barely detectable.

3 p values between non-PHN and PHN infected cells.

The ND7/23-Nav1.8 cell line harbours exogenous copies of the mouse *Scn*10 cDNA encoding the TTX-r Nav1.8 sodium ion channel which is active in DRG, and alteration in its activity is closely associated with neuropathic pain in the rat VZV PHN model [15], and in both inflammatory and visceral pain [13]. In addition to the *Nav*1.8 message species, RT.PCR confirmed abundant levels of *Nav*1.6 and *Nav*1.7 mRNAs compared to the background activity of the *Nav*1.3 species in non-infected cells. Interestingly, the expression levels of both *Nav* 1.6 and *Nav* 1.7, but not *Nav* 1.8, were significantly increased after infection with PHN but not non-PHN viruses. This finding suggested that alteration in the activity of the two TTX-s sodium channels Nav 1.6 and/or Nav 1.7 may have been contributing to our observations. This cell line was chosen for study rather than primary sensory neuronal cultures because of its expression of multiple sodium channel genes and the absence of a virally-induced cpe the presence of which would have made blind electrophysiological analysis problematic. We could not identify an additional or alternative *in vitro* model system that was as appropriate as the one used here. When the electrophysiological recordings were repeated in the presence of TTX in order to indicate which type of sodium ion channels were mediating the observed ion current increases, we found that this procedure abrogated the effect. This clearly demonstrated that TTX-s fast sodium ion channels may be candidates responsible for the observed current increases such as the Nav 1.6 and Nav 1.7 ion channels and not, somewhat surprisingly, the slow TTX-r Nav 1.8 species. The Nav 1.7 species is present in DRG and sympathetic neurons [13] and is recognised as being involved in neuropathic pain conditions. Mutations of the human *Nav* 1.7 ion channel gene can cause neuropathic pain syndromes in both inherited erythromyalgia and paroxysmal extreme pain syndrome [30], and have recently been implicated in the pathogenesis of small fiber neuropathy [31]. The Nav 1.6 channel is present at the nodes of Ranvier and has been shown to be dysregulated in DRG neurons in an experimental rat model of diabetes [32].

These data show several differences from those previously reported in other studies using the rat VZV pain model [19]–[23]. First, we observed modest but significant increases in *Nav* 1.6 and *Nav* 1.7 message levels but not in the *Nav* 1.8 or *Nav* 1.3 species. This contrasts with the findings of Garry *et al* [15] in which VZV infection in rats resulted in increases of both Nav 1.3 and Nav 1.8 proteins in the DRG using immunoblotting (levels in the activity of the Na 1.6 and Na 1.7 channels were not reported in that *in vivo* study). While the reasons for these differences are not known, it should be appreciated that the two model systems are very different-one *in vitro* and the other *in vivo*, and it would not have been feasible to perform voltage clamping experiments in the latter tissues. Second, in all the rat VZV PHN model studies, it had been found that both wild type or clinical isolates were capable of inducing key measurements of pain behaviour with mechanical allodynia and/or alteration of thermal thresholds leading to thermal hyperalgesia [15], [19]–[23]. By contrast, in our *in vitro* system PHN-associated VZV rather than non-PHN associated VZV increased sodium ion channel activity. It was clearly not possible to look at whole animal pain responses in our study and we recognise that we did not measure pain behaviour or pain thresholds that limits the interpretation of our findings. However, we do not believe that this in itself invalidates either the technical validity or the potential significance of our observations which approached the problem in a very different way. While host factors are clearly major determinants of PHN development in humans, it is certainly possible that the particular viral stain may also play a role in this condition. Nevertheless we recognise the discrepancies between the two models the causes of which are as yet unclear. Another difficulty arises when one considers these findings in relation to our previously reported studies of the VZV transcriptome in PHN and non-PHN VZV [25]. The microarray approach adopted there showed that viral gene expression within a group (PHN versus non-PHN) varied as much, or more, than the viral transcriptome between these groups. While it is difficult to reconcile these two findings, we believe that if there are viral gene changes that may be responsible for alterations in sodium ion channel activity, then they are probably subtle and may well have been below the threshold of sensitivity of microarray studies. A complex series of viral chimera experiments would probably be required to locate any genetic changes that may be responsible.

The failure of acyclovir treatment to abrogate the sodium ion channel activity increases in PHN-infected cells indicates that these effects were not dependent on viral replication, or, at least, not on late events in the viral cycle [33]. However, the former interpretation seems highly likely because the sonicated cell free VZV isolates did not produce infectious virus in the ND7/23-Nav1.8 cell line and, though VZV transcripts, including those transcribed from late viral genes, were detected in the cell line during 72 hr of infection, these levels were only 10–25% of those detected in the permissive, infected MeWo cells. While the acyclovir experiments were predictable from previous in vivo studies [20], [21], as well as the absence of obvious viral replication, these were controls that needed to be performed as part of the system validation. Consistent with the known human and primate tropism of VZV, the viral isolates only produced a limited infection of the cell line similar to VZV infected Chinese hamster ovary cells where both immediate-early and late gene transcription were detected without infectious virus production [34], and also consistent with an earlier study on VZV infection of mouse neuro-2A cells which failed to produce infectious virus but were transcript positive [35]. We do not currently know how VZV produces the observed phenotypic effect on ion channel currents and further studies will be required to unravel the detailed molecular events involved. However, there is a precedent for a herpes virus (Herpes simplex virus-1) latency-associated transcript (LAT) influencing the accumulation of host cell transcripts encoding apoptotic regulatory proteins [36], so an analogous mechanism may be relevant here. While we did not detect VZV proteins in our system, it should be noted that alphaherpes viruses, including VZV and herpes simplex virus (HSV) can lead to a common neuronal response resulting in numerous gene expression changes in ganglia [37]. Further, HSV-1 latency-associated transcript infection of cultured trigeminal neurons results in increased levels of the neuropeptide Substance P which is known to be associated with neuropathic pain [38] and sodium ion channel proteins have been detected in the rat VZV pain model [15]. Such findings raise the issue as to whether many of the numerous gene expression changes seen in the DRG and TG may result from virus infection, but that many of these changes may reflect the fact that a pain response exists rather then being the cause of that change in pain behaviour resulting in PHN. It is also possible that increases in calcium channels may play a role in PHN [15] and this is an aspect of neuropathic pain that we plan to investigate in future studies.

Clearly, the findings reported here need to be extended to a larger cohort of patients, but if confirmed, then they could have relevance to one possible mechanism of PHN. We also cannot exclude the possibility that our *in vitro* findings may have nothing to do with long-term changes in DRG neurons in PHN patients. Whether or not our *in vitro* findings represent an epi-phenomenon or a pathogenetically relevant finding remains to be elucidated.
